# Spontaneous Left Cardiac Isomerism in Chick Embryos: Case Report, Review of the Literature, and Possible Significance for the Understanding of Ventricular Non-Compaction Cardiomyopathy in the Setting of Human Heterotaxy Syndromes

**DOI:** 10.3390/jcdd6040040

**Published:** 2019-11-08

**Authors:** Jörg Männer

**Affiliations:** Group Cardio-Embryology, Institute of Anatomy and Embryology UMG, Georg August University Göttingen, D-37075 Göttingen, Germany; jmaenne@gwdg.de; Tel.: +49-551-39-7032

**Keywords:** heterotaxy syndromes, cardiac isomerism, chick embryo, *Pitx2*, proepicardium, non-compaction cardiomyopathy

## Abstract

The outer shape of most vertebrates is normally characterized by bilateral symmetry. The inner organs, on the other hand, are normally arranged in bilaterally asymmetric patterns. Congenital deviations from the normal organ asymmetry can occur in the form of mirror imagery of the normal arrangement (situs inversus), or in the form of arrangements that have the tendency for the development of bilateral symmetry, either in a pattern of bilateral left-sidedness (left isomerism) or bilateral right-sidedness (right isomerism). The latter two forms of visceral situs anomalies are called “heterotaxy syndromes”. During the past 30 years, remarkable progress has been made in uncovering the genetic etiology of heterotaxy syndromes. However, the pathogenetic mechanisms causing the spectrum of cardiovascular defects found in these syndromes remain poorly understood. In the present report, a spontaneous case of left cardiac isomerism found in an HH-stage 23 chick embryo is described. The observations made in this case confirmed the existence of molecular isomerism in the ventricular chambers previously noted in mouse models. They, furthermore, suggest that hearts with left cardiac isomerism may have the tendency for the development of non-compaction cardiomyopathy caused by defective development of the proepicardium.

## 1. Introduction

Except for a few species (e.g., flatfish), the outer shape of vertebrates is normally characterized by bilateral symmetry. The inner organs, on the other hand, normally are arranged in bilaterally asymmetric patterns, which are of special importance for the normal function of the cardiovascular system of lung-breathing vertebrates.

Congenital deviations from the normal organ asymmetry can occur in the form of mirror imagery of the normal arrangement-so-called “situs inversus”, or in the form of arrangements that have the tendency for the development of bilateral symmetry, either in a pattern of bilateral left-sidedness (left visceral isomerism) or bilateral right-sidedness (right visceral isomerism). The latter two forms of visceral situs anomalies are usually classified as “visceral heterotaxy syndromes” or “heterotaxy syndromes” [1]. They are typically associated with complex cardiovascular malformations [2].

During the past 30 years, remarkable progress has been made in uncovering the genetic etiology of visceral heterotaxy syndromes [2,3]. However, the pathogenetic mechanisms causing the spectrum of cardiovascular defects found in these syndromes still remain poorly understood.

Studies on chick embryos have significantly contributed to the discovery of the genetic and molecular background of the normal as well as abnormal development of the visceral situs of vertebrates [3]. In the present report, a spontaneous case of left cardiac isomerism found in an HH-stage 23 chick embryo is described. The observations made in this case suggest that hearts with left cardiac isomerism may have the tendency for the development of non-compaction cardiomyopathy caused by defective development of the proepicardium.

## 2. Materials and Methods

The abnormal chick heart presented in this report was a lucky find. It belonged to a series of embryonic chick hearts (n = 15) that were prepared for a left-right lineage tracing study by a PhD student. For this study, fertilized chicken eggs (White Leghorn) were obtained from the Georg-August University research farm and incubated at 38 °C and 75% humidity. Chick embryos of normal external morphology were fixed for whole-mount in situ hybridization (ISH) with the left-lineage marker *Pitx2* [4] at various developmental stages (incubation days 3 to 5; stages 15 to 25 according to Hamburger and Hamilton (HH), [5]). Eggs and embryos were handled in accordance with the Declaration of Helsinki and the local animal protection laws, which did not claim the approval of the study by an institutional review board or ethics committee. Prior to fixation, the hearts were arrested in an end-diastolic state [6]. After fixation, a tissue block consisting of the heart and the mediastinum was dissected free from the embryo. Whole-mount ISH of the tissue blocks was carried out according to established protocols [6]. Subsequent to whole-mount ISH, the stained specimens were critical point dried in order to facilitate stepwise dissection of dried heart specimens with fine tungsten needles in alternate with “gross morphological” analyzes under a dissection microscope. For histological analyzes of the abnormal heart specimen, the dried specimen was re-transferred into a fluid medium (methyl benzoate) and prepared for histological analyses according to established protocols [6].

## 3. Results

**Figure 1:**
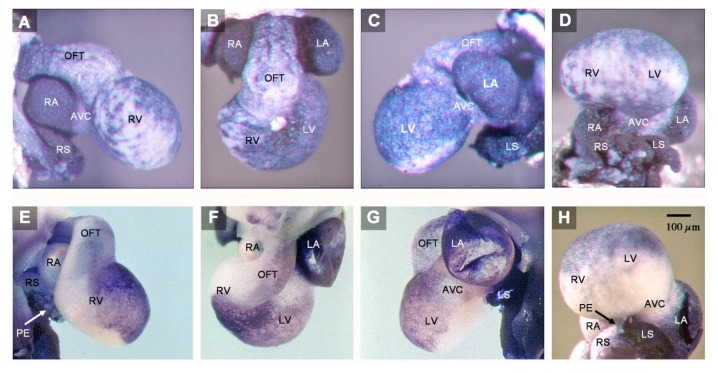
These microphotographs depict the outer shape and *Pitx2* expression pattern of the abnormal HH-stage 23 heart with left cardiac isomerism (**A**–**D**) and of a normal HH-stage 23 embryonic chick heart (**E**–**H**). Specimens are shown in right lateral views (**A**,**E**), cranio-ventral views (**B**,**F**), left lateral views (**C**,**G**), and caudal views (**D**,**H**). The abnormal heart shows a general *Pitx2* expression (blue staining), whereas the normal heart expresses *Pitx2* only in those areas that are derived from the left heart fields. The abnormal heart, furthermore, shows a tendency for bilaterally symmetric development of its components, which is especially prominent at the sinus venosus and the atria. The left and right atrium of the abnormal heart have almost the same size and shape (**B**), whereas there is a marked difference in size between the two atria of the normal heart (**F**). Note also that the abnormal heart lacks a proepicardium-derived tissue bridge (**A**,**D**), which normally bridges the pericardial cavity between the right sinus horn and the dorsal wall of the embryonic ventricles (**E**,**H**). The proepicardium is a morphological marker for right-sidedness. Abbreviations: AVC = atrioventricular canal; LA = left atrium; LS = left sinus horn; LV = embryonic left ventricle; OFT = outflow tract; PE = proepicardium-derived tissue bridge; RA = right atrium; RS = right sinus horn; RV = embryonic right ventricle.

The abnormal heart under discussion was obtained from a four-day-old chick embryo (HH-stage 23). The heart was first noticed as representing an unusual specimen after whole-mount ISH with the *Pitx2* probe. The specimen differed from all other hearts of the series by intense blue staining of its entire wall (Figure 1A–D). The normal hearts of the series showed the typical *Pitx2* expression pattern, which was characterized by blue staining of only the left heart field-derived portions of the heart (Figure 1E–H). Having noticed the unusual staining pattern of the heart specimen, its external morphology and *Pitx2* expression pattern were carefully analyzed under a dissection microscope and compared with the morphology and *Pitx2* expression pattern of normal HH-stage 23 heart specimens. The analysis of the external shape was followed by the analysis of the internal morphology on serial histological sections. Microscopical analyzes disclosed several abnormalities, which are presented here in sequential segmental order along the physiological flow path, starting at the venous heart pole.

**Figure 2:**
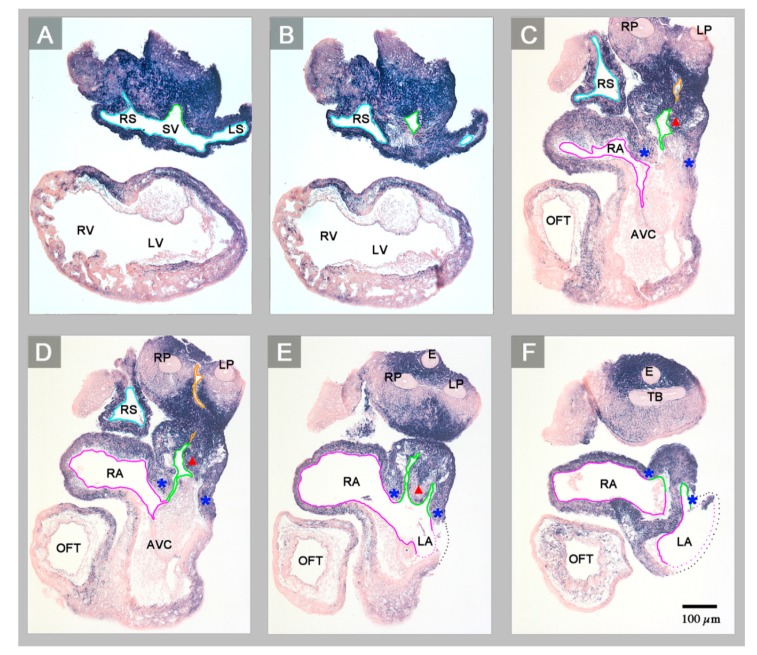
Starting at the level of the confluence of the systemic veins (sinus venosus), this sequence of transverse histological sections depicts the *Pitx2* expression pattern and the veno-atrial connections of the abnormal HH-stage 23 heart with left cardiac isomerism. *Pitx2* is expressed in the entire wall of the heart (blue staining). The sinus venosus (endothelial lining marked in light blue) drains to a narrow midline canal (**A**,**B**) that belongs to the atrial inflow component (endothelial linings marked in green). The atrial inflow component is connected to both atria (endothelial linings marked in magenta) and incompletely divided into left and right portions by a rudimentary interatrial septum (red arrowhead marks the crest of the septum) (**E**,**F**). The atrial inflow component is demarcated from the rest of the atria by two myocardial folds (marked by blue asterisks). Note that the common pulmonary vein (endothelial linings marked in orange) is connected to the right portion of the atrial inflow component (**C**,**D**). Abbreviations: E = esophagus; LP = left lung bud; TB = tracheal bifurcation; RP = right lung bud; SV = sinus venosus; other abbreviations as used before.

**Figure 3:**
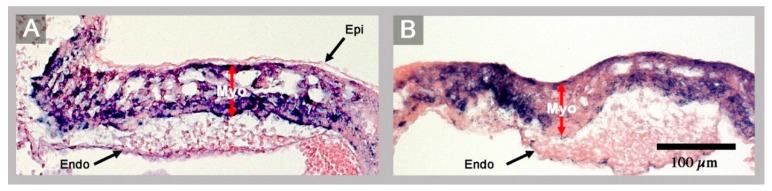
These histological sections show the ventricular wall architecture of a normal HH-stage 23 embryonic heart (**A**) and of the abnormal HH-stage 23 heart with left cardiac isomerism (**B**). (**A**) The normal ventricular wall consists of three layers: the epicardium (Epi), the myocardium (Myo), and the endocardium (Endo). (**B**) The ventricular wall of the abnormal heart lacks the epicardium. Red arrows mark the thickness of the myocardial layer of the ventricular wall.

**Figure 4:**
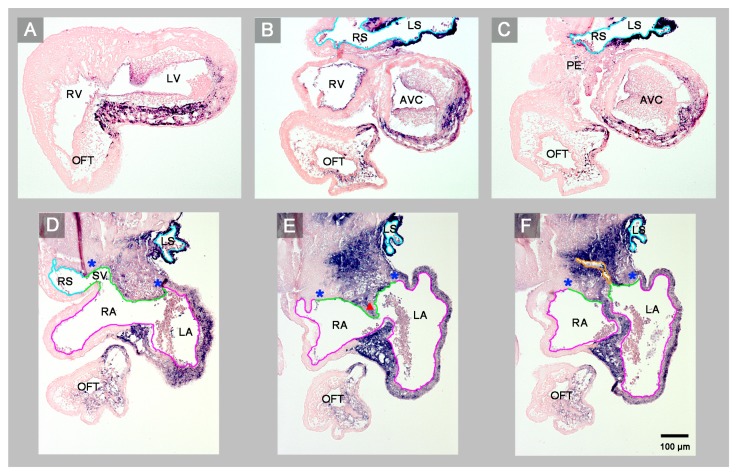
Starting at the level of the embryonic ventricles, this sequence of transverse histological sections depicts the *Pitx2* expression pattern and the venoatrial connections of a normal HH-stage 23 embryonic chick heart. *Pitx2* is expressed only in those portions of the heart that are derived from the left heart fields (left sinus horn (**B**,**C**), left atrium and interatrial septum (**D**–**F**), ventral wall of the atrioventricular canal and ventricles (**A**–**C**)). The sinus venosus drains to the right atrium via the right portion of the atrial inflow component (**D**). The common pulmonary vein (endothelial lining marked in orange) drains to the left atrium via the left portion of the atrial inflow component (**F**). Note that the ventral wall of the right sinus horn harbors the proepicardium-derived tissue bridge (**C**). Color code for endothelial linings as used before. Abbreviations as used before.

(1) The confluence of the systemic veins (sinus venosus) of HH-stage 23 hearts normally is characterized by bilateral asymmetry. The sinus venosus normally drains exclusively to the right-sided atrium and the *Pitx2* expression normally is confined to the wall of the left sinus horn. The *Pitx2* negative right sinus horn harbors a *Pitx2* negative proepicardium-derived tissue bridge, which connects its ventral wall with the dorsal wall of the ventricular bend (Figure 1E,H). The left sinus horn of chick embryos normally does not form a proepicardium-derived tissue bridge [6,7]. In contrast to the normal situation, the sinus venosus of the abnormal HH-stage 23 chick heart showed a bilaterally symmetric arrangement with respect to molecular expression patterns as well as morphology. *Pitx2* was expressed in the walls of the left as well as the right sinus horn, and both sinus horns did not show any trace of a proepicardium-derived tissue bridge (Figure 1A,C,D and Figure 2A,B). The absence of the proepicardium-derived tissue bridge was associated with the defective formation of the epicardium (Figure 3). The left and right sinus horns were of the same size and drained to a narrow midline canal (Figure 2A,B). This canal was continuous with a dorsal component of the developing atria (Figure 2A–E), which we called the “atrial inflow component” [8].

(2) The atrial segment of HH-stage 23 hearts normally is characterized by bilateral asymmetry. There is a marked difference in size between the two atria (the right atrium is smaller than the left) and *Pitx2* expression is confined to the left atrium (Figure 1E–H). The sinus venosus is connected to the right atrium via the right portion of the atrial inflow component, while the stem of the pulmonary veins is connected to the left atrium via the left portion of the atrial inflow component (Figure 4). In contrast to the normal situation, the atrial segment of the abnormal HH-stage 23 chick heart showed bilateral symmetry. Both atria were of almost the same size and *Pitx2* was expressed in the left as well as the right atrium (Figure 1A–D). The sinus venosus drained via a narrow midline canal to the atrial inflow component, which was connected to both atria (Figure 2A–E). The stem of the pulmonary veins was abnormally connected to the right portion of the atrial inflow component (Figure 2C,D).

(3) The atrioventricular canal (AVC), the primitive left and right ventricles, and the outflow tract (OFT) of early embryonic hearts normally undergo a process of ventricular looping morphogenesis, which changes the original form and position of these embryonic heart segments [9]. Ventricular looping generates a helically wound bend whose outer curvature normally points toward the right body side (ventricular D-loop). This sets the scene for the development of the normal topographical relationship of the two ventricles. Looping toward the left body side (ventricular L-looping) is abnormal and sets the scene for the development of a ventricular topology that is the mirror image of the normal one. In the setting of human cardiac isomerism, ventricular looping is said to occur in a random fashion, which means that only 50% of the cases develop ventricular D-loop topology while the remaining 50% develop ventricular L-loop topology [10]. The abnormal HH-stage 23 chick heart presented here had a ventricular D-loop. The configuration and *Pitx2* expression pattern of this D-loop, however, differed from the normal one (Figure 1A,B). Compared to normal HH-stage 23 hearts, the ventricular D-loop of the abnormal heart specimen was mainly characterized by an abnormal tendency for ventral positioning of the embryonic right ventricle and straightening of the OFT. *Pitx2* was abnormally expressed in the entire wall of the AVC, primitive ventricles, and OFT (Figure 1A–D and Figure 2). This means that *Pitx2* expression occurred in both the ventral and dorsal walls of the AVC, the primitive ventricles and OFT. Thereby, the ventral and dorsal regions of ventricular *Pitx2* expression were separated from each other by a zone of less intense *Pitx2* expression, which ran along the outer curvature of the ventricular segment and, therefore, seemed to correspond to the original ventral midline of the heart tube. (Figure 1A,C,D). In normal HH-stage 23 chick hearts, ventricular *Pitx2* expression is confined to the ventral walls of the embryonic ventricles and to the left-ventral part of the OFT only (Figure 1E–H and Figure 4A–C).

## 4. Discussion

The abnormal HH-stage 23 embryonic chick heart presented in this report showed a tendency for bilaterally symmetric-“isomeric”-formation of all of its components. This tendency was especially prominent at its venous pole (sinus venosus, atria). Here, the bilateral expression of *Pitx2*, which is a molecular marker for left-sidedness [11], and the bilateral absence of a proepicardium-derived tissue bridge, which is a morphological marker for right-sidedness in avian embryos [6,7,12], indicate the presence of bilateral left-sidedness. Bilateral symmetry was also indicated by the fact that both atria were of almost the same size and morphology. Downstream to the atria, the discovery of a tendency for bilateral symmetry is usually made difficult by the process of ventricular D-looping, which converts the original positions of the left and right halves of the AVC, primitive ventricles and OFT into ventral and dorsal positions, respectively [9,11]. In these components of the abnormal embryonic chick heart, the presence of bilateral left-sidedness was indicated by the abnormal *Pitx2* expression in both the ventral and dorsal walls.

The tendency for development of bilateral left-sidedness is usually found in a subset of the visceral heterotaxy syndrome that has been termed “polysplenia syndrome” [13], “left isomerism” [13,14], “left cardiac isomerism” [15], “left atrial isomerism” [16], or “left isomerism of the atrial appendages” [1,17,18]. The abnormal HH-stage 23 embryonic chick heart presented in this report, therefore, may be diagnosed as having left cardiac isomerism. This diagnosis is furthermore supported by the fact that the abnormal embryonic chick heart had striking hypoplasia of its systemic venous component (sinus venosus) in combination with anomalous pulmonary venous drainage to the right-sided atrium (Figure 2A–D). Such features are typically found in human hearts with left cardiac isomerism as well as in mouse models for this subset of the visceral heterotaxy syndrome [18,19,20]. Previous data from mice disclosed a correlation between the isomeric expression of *Pitx2* and the occurrence of the double outlet from the right ventricle (DORV) [11]. The present case of an abnormal embryonic chick heart with the isomeric expression of *Pitx2*, unfortunately, could not be assessed for the presence of DORV since it was fixed prior to stages when the phenotype of DORV first becomes apparent (incubation days 8/9, ~HH-stage 32–34).

Patho-morphological studies on cases of visceral heterotaxy have provided evidence for the existence of morphological isomerism at the level of the atrial chambers (isomerism of the atrial appendages), while the possible existence of ventricular isomerism has been questioned for a long time [10]. The analysis of *Pitx2* expression facilitates the identification of the molecular left-right identities in the atrial as well as ventricular compartments of embryonic hearts. Using this approach in a study on embryonic mice with heterotaxy, Campione and co-workers were able to reveal the existence of molecular isomerism not only at the level of the atrial chambers but also at the level of the ventricular chambers [11]. They have, furthermore, shown that molecular left isomerism of the atrial and ventricular chambers not always occur in combination, suggesting that cardiac *Pitx2* expression may be controlled by compartment-specific activation pathways. The present case of an abnormal HH-stage 23 embryonic chick heart displayed molecular left isomerism at the level of the atrial as well as ventricular chambers. The present data thus confirm the existence of molecular ventricular isomerism in chick embryos. Due to the fact that the present data are derived only from a single spontaneous case, however, they cannot provide information about the correlation between the occurrence of atrial and ventricular isomerism in chick embryos.

Over the past three decades, remarkable progress has been made in the elucidation of the genetic and molecular control of the development of the visceral situs of vertebrates [2,3]. This progress led to the generation of several genetically modified mouse models for visceral heterotaxy syndromes with left- [15,21,22] or right cardiac isomerism [23,24,25,26,27,28,29]. This list of mouse models for visceral heterotaxy is completed by mouse strains carrying spontaneous mutations of situs-relevant genes [17,30] as well as by teratogen-induced mouse models [19,31,32]. In view of this fact, the question may arise as to why I do report on this single case of an abnormal embryonic chick heart? Regarding this question, I should note that studies on chick embryos have significantly contributed to the discovery of the genetic and molecular background of the normal as well as abnormal development of the visceral situs of vertebrates [3]. Furthermore, the chick embryo is a well-established model for studying the normal as well as abnormal embryonic development of the heart of higher vertebrates [33,34]. Each animal model for human disease has its own specific advantages and drawbacks. We, therefore, should keep in mind that studying the etiopathogenesis of heterotaxy syndromes only on mouse models might hamper the discovery of some pathogenetic processes acting in human heterotaxy syndromes. Studies on chicken models for cardiac isomerism may help to discover pathogenetic mechanisms that possibly cannot be easily uncovered in mouse models for human heterotaxy syndromes. Unfortunately, however, the currently available experimental chicken models for visceral heterotaxy syndromes do not facilitate analyzes of abnormal cardiogenesis beyond the early stages of cardiac looping morphogenesis. Thus, at the present time, the possibility to analyze the hearts of chicken with visceral heterotaxy syndromes at advanced stages of cardiogenesis depends on the availability of spontaneous cases, only. Such cases are rare findings. A careful search for reports on spontaneous cases of cardiac isomerism in chicken disclosed only two reports written in French [35,36], but no report written in English or other languages. In the two papers written in French, Dor and co-workers have documented six spontaneous cases of cardiac isomerism found in four-day-old chick embryos. Unfortunately, these authors did not distinguish between left and right cardiac isomerism. They simply classified all of their cases as “atrioventricular situs ambiguus” on the basis of pathomorphological analyzes using scanning electron microscopy. Since these specimens were analyzed before the discovery of the molecular markers for left-sidedness, there was no molecular evidence supporting the correct assignment of these cases either to the group of left cardiac isomerism or right cardiac isomerism. Thus, the present case seems to be the first example of an abnormal embryonic chick heart displaying molecular and morphological left isomerism at an advanced stage of embryonic cardiogenesis. A comparison of the morphological features of this case with those published by Dor and co-workers shows that at least one of their cases (see Figure 58 in [36]) seems to fit the diagnosis left cardiac isomerism.

It is the question of whether the present case may have any relevance for the understanding of human congenital heart defects in the setting of visceral heterotaxy syndromes? The answer to this question may be found at the sinus venosus of this heart, which shows a bilateral absence of a proepicardium-derived tissue bridge (Figure 1A,C,D). The proepicardium (PE) is a primarily extracardiac population of embryonic progenitor cells that normally provides the epicardial mesothelium, the subepicardial and intramyocardial fibroblasts, and several cell lineages of the coronary blood vessels [7]. It forms in the area of the sinus venosus, where it is found as a cauliflower-shaped accumulation of villous protrusions of the pericardial coelomic epithelium. The first PE-derived cells normally reach the originally naked myocardial surface of the embryonic heart during the second phase of cardiac looping (S-looping; HH-stage 17 in chick embryos). They form the primitive epicardium, which then provides mesenchymal cells that colonize the subepicardial and myocardial wall layers where they differentiate into fibroblasts, coronary smooth muscle cells, and coronary endothelial cells. In amphibian, reptilian and avian embryos, the transfer of PE cells to the developing heart normally is accomplished via a secondary tissue bridge, which is formed by the firm attachment of the PE to the dorsal surface of the developing ventricles. The PE arises from bilaterally paired anlagen [6]. In avian and amphibian embryos, PE development normally shows a visible pattern of bilateral asymmetry. Here, only the right-sided PE anlage normally undergoes a remarkable growth in size and becomes the functioning PE that finally forms a PE-derived tissue bridge. The left-sided PE anlage normally remains in a rudimentary state and does not significantly contribute to heart development [6,37]. Thus, the PE and the PE-derived tissue bridge of chick embryos are features of morphological right-sidedness. The complete absence of a PE-derived tissue bridge found in the present chick heart with left cardiac isomerism, thus, can be interpreted as a feature of bilateral left-sidedness. The consequences of the defective formation of the PE have been demonstrated by experimental studies on chick embryos [38]. The experimentally induced loss of the PE or the prevention of the formation of the PE-derived tissue bridge leads to a severe delay in the formation of the epicardium combined with the deficient formation of the subepicardial and intramyocardial connective tissue, and coronary vessel defects. The experimentally induced “loss of PE function syndrome”, additionally, includes a severe growth defect of the compact layer of the ventricular myocardium resembling the so-called “ventricular non-compaction cardiomyopathy” found in human beings. Corresponding observations were made in mice with embryonic epicardial defects and it has been shown that the non-compaction cardiomyopathy found in epicardium-deficient embryos resulted from the lack of trophic signals normally provided by the embryonic epicardium [39,40]. Based on these experimental data, it can be stated that the abnormal embryonic chick heart presented here, which lacks a PE-derived tissue bridge and the normal epicardial covering of its ventricular myocardium (Figure 1A,D; Figure 2A,B; and Figure 3), shows an early stage of development of the loss of PE function syndrome. It is evident that this heart would have developed the full loss of PE function syndrome if the embryo were allowed to survive up to developmental stages when the myocardial non-compaction phenotype usually becomes apparent (~HH-stages 29/30). Thus, the present case of an embryonic chick heart with left cardiac isomerism strongly suggests that chicken heterotaxy syndromes with left cardiac isomerism have the tendency for the development of the loss of PE function syndrome including non-compaction cardiomyopathy. Chicken heterotaxy syndromes with right cardiac isomerism, on the other hand, should not have this tendency, since it is to be expected that the affected embryos have the tendency for the development of two functioning PEs. Based on these reflections, it is tempting to speculate that human heterotaxy syndromes with left cardiac isomerism may also have the tendency for the development of non-compaction cardiomyopathy caused by defective development of the proepicardium. A web-based search for articles reporting on the occurrence of non-compaction cardiomyopathy in the setting of human heterotaxy syndromes, indeed, disclosed several cases with left cardiac isomerism but not a single case with right cardiac isomerism [41,42,43,44,45].

With respect to the isomerism-induced loss of PE function, I should note that the development of the PE in mammalian embryos differs from the situation found in amphibian and avian embryos. Mammalian embryos do not develop a morphological asymmetry of their functioning PE. The PE of mammalian embryos normally is formed by the union of the left and right PE-anlagen, and both halves of the PE seem to provide equal amounts of PE-derived cells to the developing heart [6]. On the first view, this fact seems to conflict with the speculation described above. We should take into account, however, that the presence of morphological symmetry not necessarily means that the two halves of the mammalian PE harbor functionally equivalent cell populations. It is conceivable that the molecular signals that normally control body sidedness might also cause side-specific differences in the composition of PE cell populations, so that normally only the right halve of the mammalian PE may harbor the cell population that will later provide trophic signals to the compact layer of the developing myocardium.

It should finally be noted that isomerism-induced loss of PE function is not the only hypothesis providing an explanation for the association between left cardiac isomerism and non-compaction cardiomyopathy found in human patients. It is conceivable that this association might be caused by functional defects in a signaling pathway that is involved in the control of left-right patterning as well as in the control of myocardial proliferation. In this respect, one of the currently most attractive candidates may be the retinoic acid pathway (RA). RA signaling is involved in the determination of body sidedness [22] as well as in the control of the growth of the embryonic myocardium [39,40]. Abnormal RA signaling during the early development of mouse embryos causes heterotaxy syndromes, including left cardiac isomerism [19], and mouse embryos lacking the RA receptor RXRα develop a severe growth defect of the compact layer of the ventricular myocardium resembling non-compaction cardiomyopathy [46].
